# Attentional state and brain processes: state-dependent lateralization of EEG profiles in horses

**DOI:** 10.1038/s41598-018-28334-9

**Published:** 2018-07-05

**Authors:** C. Rochais, M. Sébilleau, M. Menoret, M. Oger, S. Henry, M. Hausberger, H. Cousillas

**Affiliations:** 1 Université de Rennes, UMR 6552 −Laboratoire Ethologie Animale et Humaine-EthoS-, CNRS, Université de Caen-Normandie, Station Biologique, 35380 Paimpont, France; 2 Université de Rennes, UMR CNRS 6552 -Laboratoire Ethologie Animale et Humaine-EthoS- CNRS, Université de Caen-Normandie, Campus de Beaulieu, 263 avenue du général Leclerc, 35042 Rennes cedex, France; 3 Université de Rennes, UMR CNRS 6164, IETR Institut d’Electronique de Rennes, Campus de Beaulieu, Avenue du Général Leclerc, 35042 Rennes cedex, France; 4 CNRS- UMR 6552, −Laboratoire Ethologie Animale et Humaine-EthoS-, Université de Rennes 1, Université de Caen-Normandie, 263 avenue du Général Leclerc, 35042 Rennes Cedex, France

## Abstract

Lateralization of brain functions has been suggested to provide individuals with advantages, such as an increase of neural efficiency. The right hemisphere is likely to be specialized for processing attention for details and the left hemisphere for categorization of stimuli. Thus attentional processes actually may underlie lateralization. In the present study, we hypothesized that the attentional state of horses could be reflected in the lateralization of brain responses. We used *i)* a recently developed attention test to measure horses’ visual attentional responses towards a standardized stimulus and *ii)* a recently developed portable EEG telemetric tool to measure brain responses. A particular emphasis was given to the types of waves (EEG power profile) and their side of production when horses were either attentive towards a visual stimulus or quiet standing. The results confirmed that a higher attentional state is associated with a higher proportion of gamma waves. There was moreover an interaction between the attentional state, the hemisphere and the EEG profile: attention towards the visual stimulus was associated with a significant increase of gamma wave proportion in the right hemisphere while “inattention” was associated with more alpha and beta waves in the left hemisphere. These first results are highly promising and contribute to the large debate on functional lateralization.

## Introduction

Lateralization of brain functions appears as a key property of most vertebrates^1^ as well as of invertebrates^2^. Despite species differences, some basic patterns of lateralization seem to exist in terms of attention, emotion^1^, or nature of the cognitive task performed^3–5^.

The left hemisphere is supposed to be specialized in categorizing information and responds more to features that are invariant and repeated, while the right hemisphere responds more to novel events and the expression of intense emotional states such as aggression, escape behaviour and fear^3,6^. Functional brain asymmetry may be affected by age (*e.g*. in humans:^7^, reproductive status (*e.g*. in frogs:^8^) and gender (*e.g*. in humans:^9^), and some authors suggest that brain lateralized responses could be modulated by the subject attentional state even at the level of a few seconds^10^.

The relative generality of brain lateralization and the result on behavioural responses suggest that lateralization does provide individuals with advantages, such as increasing neural efficiency, by avoiding redundant neural circuitry^11^ or preventing the initiation of conflicting behavioural responses^12^. Indeed, strongly lateralized individuals have been shown to have enhanced efficiency when performing dual tasks^13^ and lateralization may enhance cognition in general^14^. For instance, Rogers^15^ showed that vigilance while pecking at food was better in chicks with lateralization of visual processing than in chicks that were not so lateralized, in relation to a specialization of the right hemisphere for attention to details, and of the left hemisphere for categorization of stimuli. Advantages of brain asymmetry have been discussed in Vallortigara and Rogers (2005)^16^. For instance, stronger visual asymmetry in pigeons enhances success in visually guided foraging and hence, enhances survival^17^.

According to Andrew and Watkins^18^, one basis for the evolution of lateralization would be attention, and it would have been of survival value to have a sustained attention on the target. In a study in European starling (*Sturnus vulgaris*), George *et al*.^19^ showed that the proportion of responsive neuronal sites of birds’ brain (in the integrative part of the song system: HVC) in response to species-specific song elements was higher in the right hemisphere when the birds were awake, whereas no such lateralization was observed when the birds were anesthetized. Authors proposed that given that attention is related to wakefulness, attention processes actually underlie lateralization.

Amongst vertebrates, horses are especially interesting as they have laterally placed eyes and almost complete decussation of the optic fibres^20^. Indeed horse’s perceptual laterality has been widely evidenced in this species. Larose *et al*.^21^ found that the time spent by horses looking at a novel fear inducing object with its left eye was correlated with their level of emotionality. In another experiment where horses were confronted to different types of objects, they showed a gradient of exploration of objects according to their emotional value and a clear asymmetry in visual exploration with more use of the left eye for objects associated with negative past experiences^22^. A directional bias to the left was also found during visual vigilance behaviour (reflecting high emotional content) in feral and Przewalski horses^23,24^. Lateralization and attention may well be related in horses. For example, in experiments where whinnies of familiar, unfamiliar or group members’ horses were broadcasted, the tested horses showed a gradient of attention and laterality from familiar to unfamiliar sounds^25,26^.

Because of these elements linking attention and laterality, we hypothesized that the attentional state of horses would be reflected in the lateralization of brain responses. In the present study, we used *i)* a recently developed attention test to measure horses’ visual attentional responses towards a standardized stimulus^27^ and *ii)* a recently developed portable EEG telemetric tool to measure brain responses^28^. A particular emphasis was given to the types of waves and their side of production.

## Results

Twelve horses were individually submitted to a visual attention test (VAT,^27^) in their home stalls once on two consecutive days (Day 1 and Day 2). The VAT consisted in the display of a laser light during 5 minutes and along a standardized movement pattern on the horses’ stall door. Horses’ behaviour was videotaped and they were equipped with a telemetric portable EEG device. For each horse, relative EEG power profile was compared when it was clearly attentive towards the stimulus (*i.e*. “fixed” visual attention behaviour characterized by the exclusive use of the binocular visual field associated with a total fixity of the horse’s body, including neck, head, ears and eyes and both ears pointed towards the stimulus^27^) to when it was just quiet standing (*i.e*. horses with the eyes open, the muscles relax and the ears rotate laterally^29,30^).

All horses showed both times of attention towards the visual stimulus and times of quiet standing state (30.2 ± 5.9 and 17.3 ± 5.9 seconds attentive: 13.7 to 82.6 s and 4.8 to 53.9 at Day 1 and Day 2 respectively; 106.3 ± 51.5 and 36.1 ± 13.6 seconds in a quiet state: 4.0 to 78 s and 3.0 to 81.0 s at Day 1 and Day 2 respectively). The number of attention sequences varied between 5.0 and 34.0 (11.8 ± 2.8), lasting between 1.4 and 4.5 seconds (2.8 ± 0.3 s). Fewer subjects (N = 3) reacted towards the visual stimulus on Day 2 suggesting some habituation.

The EEG recordings were in accordance with those obtained in previous studies using external or implanted electrodes, with a clear desynchronization, low to medium voltages and slow and more rapid waves superimposed^31^. The relative part of the different waves, revealed that low frequencies (Delta, δ, and Theta, θ), characteristic of slow wave sleep state, as well as low amplitudes were less represented. Three frequency waves were over represented in all horses: the Alpha waves (α:8–12 Hz) characteristic of a relaxed state, the Bêta (β: 12–30 Hz) and the Gamma waves (γ: > 30 Hz), characteristic of higher awareness states. When horses were attentive towards the visual stimulus the proportion of gamma waves was higher (alpha = 28.6 ± 2.5%, gamma = 36.8 ± 2.5%; Wilcoxon signed rank test: T_(N=10)_ = 0, P = 0.005) and the proportion of alpha waves lower (alpha = 36.9 ± 1.3%, gamma = 28.6 ± 2.4%; T_(N=10)_ = 1, P = 0.007) than when they were in a quiet standing state. Only the proportion of beta waves did not differ according to the horse’s attentional state (attention = 34.7 ± 0.9%, quiet = 34.5 ± 1.2%: T_(N=10)_ = 26, P = 0.87). These differences were not observed anymore on the second day of testing (Wilcoxon signed rank test: T_(N=7)_ = 11, P = 0.61).

**Figure 1:**
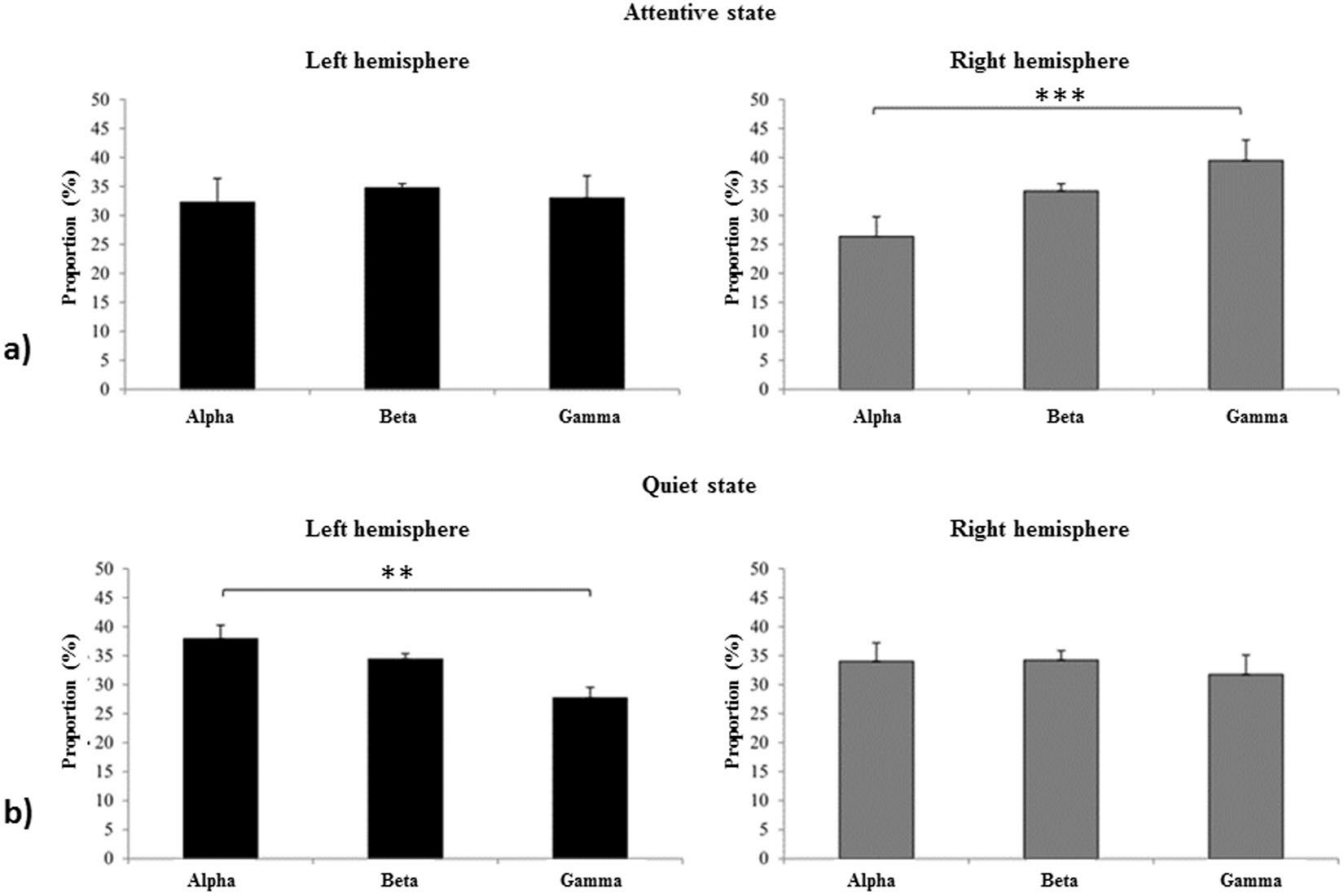
EEG wave profiles according to the attentional state and hemisphere on Day 1: (**a**) attentive state; (**b**) quiet state. The attentional state was characterized by a higher proportion of gamma waves in the right hemisphere and the quiet state by a higher proportion of alpha waves in the left hemisphere. Post-hoc Tukey test; ***P = 0.003, **P = 0.028.

There was in fact a clear interaction at D1 between attentional state, the hemisphere considered and the EEG profile (LM-M, N = 10, X^2^ = 18.3, p = 0.01). Thus, when horses were attentive towards the visual stimulus on Day 1, the proportion of alpha waves was significantly lower compared to gamma waves in the right hemisphere (RH) (*RH*: *alpha* = 26.3 ± 3.4%, *beta* = 34.2 ± 1.4%; *gamma* 39.4 ± 3.6%, *alpha/gamma*: T_(N=10)_ = 3.42, P = 0.003), while the proportion of those three waves was homogeneous in the left hemisphere (LH) (Fig. 1a). However, when horses were in a quiet standing state, the proportion of alpha waves was higher than that of gamma waves only in the left hemisphere (*LH*: *alpha* = 37.9 ± 2.4%, *beta* = 34.4 ± 0.9%; *gamma* = 27.7 ± 1.8%, *alpha/gamma*: T_(N=10)_ = −2.6, P = 0.028) (Fig. 1b). On Day 2, no significant difference was found according to horses’ state and brain laterality (LM-M: N = 7; X^2^ = 0, P = 1).

## Discussion

This approach combining behavioural testing and non-invasive EEG recordings in horses confirmed that a higher attentional state is associated with a higher proportion of gamma waves. Moreover, a clearly different pattern of lateralization appeared according to the subjects’ attentional state. Thus, attention towards a visual stimulus was associated with a significant increase of gamma wave proportion in the right hemisphere while “inattention” was associated with more alpha and beta waves in the left hemisphere.

The use of non-invasive EEG recordings during the VAT first confirmed that horses’ fixed attention (*i.e*. use of the binocular visual field associated with a total fixity of the horse’s body) is a high degree of attention as it was associated with an increase of gamma wave proportion, characteristic of higher awareness e.g.^32^. This was particularly true on Day 1, when the situation was new. Interestingly, the observed habituation towards the stimulus on Day 2 was associated with an equal distribution of all wave types. These results remind of George *et al*.^19^ study where different birds showed a same lateralization of neuronal responses towards song stimuli when they were awake but none when they were anesthetized, hence not attentive towards the stimuli anymore. The same kind of pattern was found in dogs confronted to positive human interaction: while responses were clearly observed on the first test, they disappeared with the repetition, hence with the decrease of arousal of dogs then habituated to the same stimuli^33^.

The increase of gamma wave proportion in the right hemisphere when horses were attentive confirms a lateralization of brain functions^1^ and a potential specialization of the right hemisphere for attention to details^15^. As suggested by Andrew and Watkins^18^, the earliest lateralized functions might have been attentional, with the right hemisphere picking out possible targets to which the focus attention might shift. In human, the right hemisphere is better able to sustain diffuse attention. Consistent with this, distractors are more likely to be noticed in the left hemisphere^18^. Diffuse attention should be accompanied by a wider recording of properties and spatial context. Golderg and Costa^34^ provide that the right hemisphere is better able to deal with informational complexity and to process many modes of representation within a single cognitive task.

One critical aspect of course could be the use of EEG for assessing lateralization of brain processes, given its diffuse type of recording^35^. In human, the use of head nets with multiple electrodes leads to a very generalized information. This could be of course a limiting factor in our study. However, several factors converge to confirm that our findings are indeed due to a functional lateralization: 1) the horse is a species with an almost complete decussation of the optic fibers (80–90% vs 50% in humans^36^); 2) the use of a restricted number of electrodes also restricted the chance to receive a diffuse signal; 3) the electrodes were placed on the occipital and frontal positions on each side of the forehead, which, given the high decussation, increases the chance of having hemisphere-specific responses; 4) if the information was globalized at the brain level, the clear lateralized pattern of responses would certainly not be found.

These first results combining behavioural and EEG approaches in free moving horses, are highly promising and contribute to the large debate on functional lateralization. Predicting higher attention is nevertheless important for both humans and domestic working animals. Attention is considered to underlie a variety of cognitive processes, such as learning and memory^37^, and thus can have an impact on the subject daily life. For instance, horses living under putatively challenging-to-welfare conditions show altered attentional engagement towards environmental stimuli or ‘pessimistic’ attentional and judgement biases (e.g.^38–41^). Combining attentional task such as the VAT and EEG recordings may well be a promising approach to predict affective states including not only acute emotions but also long-lasting moods.

## Material and Methods

### Ethical statement

This study was approved by the French National Ethics Committee (Animal utilisation protocol number: 33, 12-2013-12). Experiments complied with the current French laws related to animal experimentation and were in accordance with the European directive 2010/63/UE. This experiment only included behavioural observations and non-invasive interactions with the horses. Animal husbandry and care were under the management of the staff of the ‘Ecole Nationale d’Equitation’. The horses used in this experiment were not research animals.

### Subjects

The study was conducted at the ‘Ecole Nationale d’Equitation’ of Saumur (France) in July 2014, on twelve horses (N_mares_ = 5; N_geldings_ = 7), aged 7 to 10 years ( ± ES = 7.7 ± 0.3), from French Saddlebred (n = 9) and Anglo- Arabian (n = 3) breeds. They were all maintained in 3 × 3 m individual stalls with no contact allowed between neighbouring horses (*i.e*. plain wall between stalls). The stall doors had an opening on the top half that was facing indoors (*i.e*. onto the barn corridor) and the opposite wall had a window facing outdoors. The stalls were cleaned once a day; horses were fed industrial pellets three times a day (7 am, 11:30 am and 5 pm), had hay twice daily (between 8 to 9 am and between 2 to 4 pm) and had water *ad libitum* (*i.e*. automatic drinker). The horses were used for either jumping (n = 9) or eventing (n = 3) competitions. The horses went out once a day for work (±1 h/day) in the morning and remained in the stable for the rest of the day. Each horse was confronted to the visual attention test (VAT) where we compared the times when the horse was clearly attentive towards the stimulus to the time when it was just quiet standing.

### The ‘Visual Attention Test’ (VAT)

The test took place in the horse’s home stall. The experimenter entered the stall, approached the horse, equipped it with a halter and rope, fitted it with the EEG helmet and then placed it gently in front of the closed stall door. The horse was then left unrestrained (without any halter and rope) and hence able to move freely. A first phase of habituation to the experimenter was performed during 2 min: the experimenter (CR) stood immobile in the middle of the stall at 1 m distance from the stall door (facing it), on the left side and at the horse’s shoulder level. Then, during the test phase, the experimenter stood at the same location, arms along the body limiting movements to the small wrist movement when displaying the stimulus. A green light issued from a laser pointer (Laser point, PEARL®) was projected on the tested horse’s stall door (bottom half) with repeated circular clockwise and 50-cm long vertical and horizontal movements, following a predetermined order during 5 minutes by the same experimenter^27^. The test was performed once a day during 2 consecutive days (Day 1 and Day 2), once in the morning, once in the afternoon in a random order for each horse, but in any case at least 2 h after the horses’ last working session.

The tests were videotaped using two axis M1054-W cameras ® (lens 2.8 mm; horizontal field view: 84°) mounted on the left and the right top of the stall walls and by using the media-recorder® software that allowed to record and synchronize several videos. Behaviours were then encoded and synchronized with EEG recordings using “The Observer” software (Noldus®). The data were transcribed later by two observers (CR; MS; inter-observer reliability: Spearman rank order correlation R = 0.86).

### Behavioural measures

Continuous focal sampling^42^ was used to analyse the behaviours during each 5 minutes test, frame by frame (frame, 0.02 s). Behavioural analyses focused on:

(i) Fixed visual attention behaviour described in the literature as a higher degree of attention^30^ and characterized by the exclusive use of the binocular visual field associated with a total fixity of the horse’s body (including neck, head, ears and eyes) and both ears pointed towards the stimulus. The use of binocular gaze was defined as if the horse had at least one moment of ≥1 sec of gazing fixedly towards the visual stimulus^43^, with the horse facing the head (i.e. mid line of the head, between both eyes) and both eyes were oriented forwards towards the visual stimulus. The “beginning” or “end” of a gaze was defined when the horse’s head started to move into or out of the ≤45° zone, respectively^44,45^.
(ii) Standing quiet state characterized by horses with the eyes open, the muscles relax and the ears rotate laterally. This quiet state was different from observation of the environment, when the horse’s neck is held slightly higher, and from resting, when, in the standing posture, the horse often has half closed eyes and stands on only 3 legs^29,30^.

We extracted the time periods when horses were in one of those two states.

### Non-invasive EEG recordings

#### Set up

**Figure 2:**
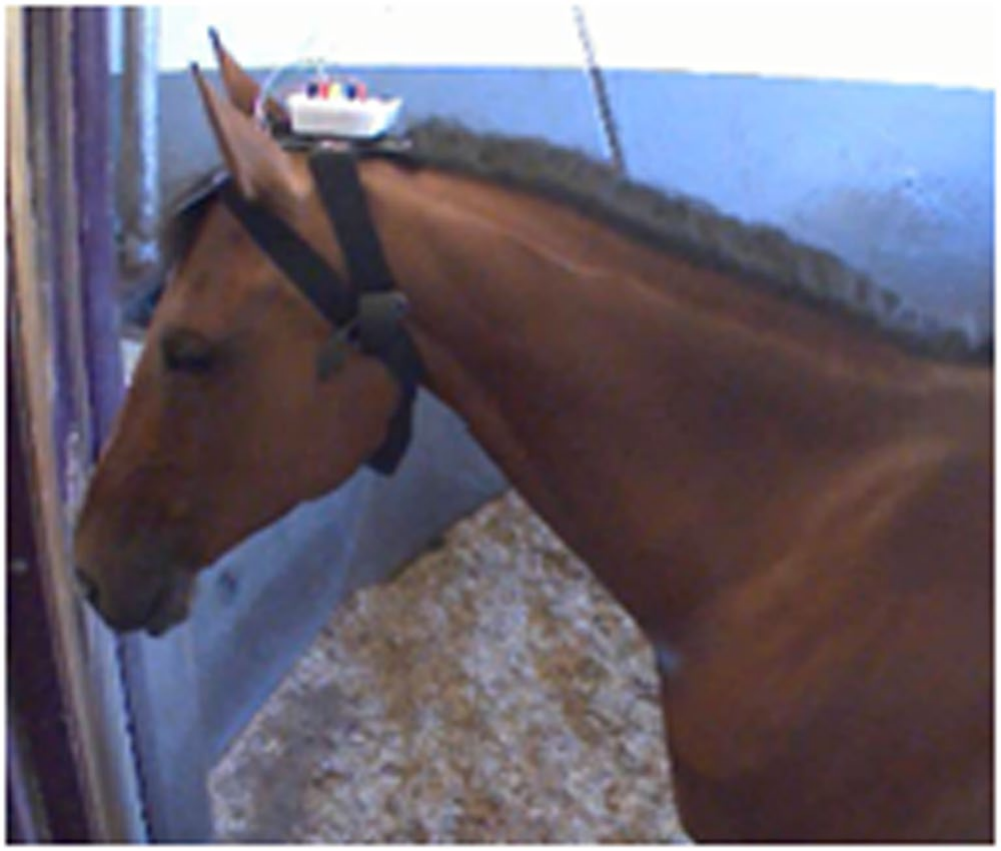
EEG device: headset placed on an attentive horse’s head.

Ten horses were available on the first test day and 7 on the second test day for EEG recordings. Five of them could be recorded on both test days. The electrophysiological recordings were performed using a homemade telemetric recording setup (L 110 mm, l 90 mm, h30mm, weighing 110 g; developed by M.O) and an ambulatory EEG headset (Fig. 2; patent # R23701WO) allowing an easy and fast positioning of 5 electrodes on the horse’s forehead over the parietal and frontal bones^28^. The electrodes were on each side of the horse’s forehead^28^ allowing recordings of the differential activity between the most occipital part of the brain and the most frontal one as well as for the left and right hemisphere separately. The ground electrode was placed on the back of the left ear (Fig. 2). The system was completed by a homemade EEG amplifier based on the 2 ways recordings “ModularEEG” from the openEEG project. This amplifier was connected to the telemetric radio transmitter. The whole telemetric recording setup was fixed on the helmet. The sampling rate was 1 kHz.

#### Data analysis

All EEG data were analysed using FieldTrip^46^, a free toolbox of Matlab 7.0 (MathWorks, Natick, MA, ®). EEG signals during attention and quiet state periods were visually inspected and large artifacts due to movements (body, head, ears) were removed. The data were then segmented into 500 ms epochs and a second visual artifact rejection procedure was performed to exclude potential remaining artefacts. A complex Fast-Fourier Transform (FFT) was applied on these 500 ms epochs. Given the difficulty to adequately identify the artifacts, for each frequency, outlying values ± 3 SD were identified and the corresponding epoch were removed from the analysis. The FFT values for each horse were averaged separately for each session and the relative EEG power values (see also^47^) of the alpha (α: 8–12 Hz), beta (β: 12–30 Hz) and gamma (γ: >30 Hz) frequency bands, characteristic of awake animals, were extracted. Wave percentage values were calculated as the proportion of the mean power values of these 3 wave types (alpha, beta and gamma).

### Statistical analysis

All statistics were performed with R v. 3.5.0 (The R foundation for statistical computing, http://www.r-project.org/). Wilcoxon tests were used to compare the EEG profiles according to the attentional state at D1 and D2 respectively. Moreover, mixed models were constructed using the lme4 and emmeans package. Normality and homogeneity of variances were assessed by inspection of residuals and Shapiro–Wilk test^48^. Differences in the relative EEG waves proportions were analyzed by performing one linear mixed model testing the proportions of different waves’ frequency as dependent variables and waves’ types (*i.e*. alpha, beta, gamma), horses’ state (*i.e*. attentive *vs* quiet), laterality (*i.e*. left *vs* right), testing day, sex and breed were specified as fixed effects and horses’ ID as random factor. AIC rank showed that the model that fit best was the one without sex and breed factor effect. Post-hoc Tukey tests were used for pairwise comparison with emmeans package. Accepted *p* level was fixed at 0.05. Descriptive statistics are means () followed by standard error (s.e.m).
